# Iodine-Doped Carbon Nitride with Enhanced Electron Delocalization as Metal-Free Sulfur Hosts for Stable Lithium–Sulfur Batteries

**DOI:** 10.3390/nano16050291

**Published:** 2026-02-25

**Authors:** Xu Yan, Ruxin Liao, Kaifu Lin, Shiman Fan, Ren He, Chaoqi Zhang, Hongbing Zhan

**Affiliations:** 1 College of Materials Science and Engineering, Fuzhou University, Fuzhou 350108, China; yanx6859@163.com (X.Y.); liaoruxin001@163.com (R.L.); linkf98@163.com (K.L.); shimanfan@outlook.com (S.F.); 2 Catalonia Institute for Energy Research–IREC, Sant Adria de Besos, 08930 Barcelona, Spain; heren940107@gmail.com

**Keywords:** carbon nitride, electron delocalization, lithium-sulfur battery

## Abstract

Suppressing the polysulfide shuttle effect and accelerating the sulfur redox kinetics remain pivotal challenges for advancing the practical viability of lithium–sulfur batteries (LSBs). In this study, an iodine-doped carbon nitride (I-CN) material was synthesized via a one-step annealing strategy and employed as a metal-free sulfur cathode host. Compared to its pristine counterpart, I-CN exhibits a substantially increased specific surface area, which facilitates the homogeneous dispersion of sulfur species. More importantly, the incorporation of iodine atoms disrupts the equilibrium of the electron cloud distribution within the CN framework, leading to enhanced electron delocalization. This electronic modulation not only significantly improves the charge transport properties of carbon nitride but also strengthens the adsorption of lithium polysulfides (LiPS) and promotes Li_2_S nucleation, thereby enabling fast and durable sulfur redox reactions. Benefiting from these synergistic effects, the S@I-CN electrode achieves high sulfur utilization, delivering an initial discharge capacity of 1341.9 mAh g^−1^ at 0.1C. Even at a high current density of 5C, a remarkable reversible capacity of 472.7 mAh g^−1^ is retained. Notably, the electrode retains 66.2% of its initial capacity after 800 cycles, demonstrating excellent long-term cycling stability. This halogen-based heteroatom doping strategy thus not only enhances the electrochemical performance of carbon nitride materials in LSBs through the rational manipulation of electron delocalization, but also offers a promising direction for the design of novel metal-free electrocatalysts in related energy conversion systems.

## 1. Introduction

Lithium–sulfur batteries (LSBs) have garnered significant attention as next-generation energy storage systems owing to their remarkably high theoretical specific capacity (1675 mAh g^−1^) and energy density (~2600 Wh kg^−1^) [1,2,3], as well as the natural abundance, low cost, and environmental benignity of sulfur [4,5]. Despite these advantages, the practical deployment of Li–S batteries remains severely constrained by several intrinsic challenges associated with the sulfur cathode. These include the insulating nature of sulfur and its discharge products (Li_2_S/Li_2_S_2_), the substantial volume changes upon cycling, and, most critically, the dissolution and shuttling of lithium polysulfides (LiPSs) between the cathode and anode [6]. This so-called shuttle effect inevitably triggers rapid capacity decay, low Coulombic efficiency, and poor cycling stability, thereby necessitating effective strategies to regulate sulfur electrochemistry and polysulfide conversion behavior [7,8].

To address these challenges, carbon-based materials have been extensively explored as sulfur host matrices due to their high electrical conductivity, chemical stability, and tunable porous structures [9]. A diverse range of carbon architectures, including porous carbons [10], carbon nanotubes [11], graphene [12], and carbon nanofibers [13], have proven effective in enhancing sulfur utilization by physically confining sulfur species and improving charge transport. However, despite these advantages, conventional carbon materials are intrinsically nonpolar, which results in weak interactions with polar LiPS molecules and thus insufficient suppression of polysulfide diffusion. Although a variety of transition-metal-based compounds have been intensively investigated in recent years to mitigate the polysulfide shuttle effect through their polar surfaces [14], these materials inevitably suffer from several intrinsic drawbacks, including relatively low specific surface areas, complicated synthesis procedures, or the potential generation of toxic byproducts. Consequently, the development of metal-free host materials via simple and scalable fabrication strategies has thus emerged as a particularly promising and worthwhile avenue for addressing the persistent challenges associated with LSBs. To address this limitation, heteroatom doping and surface functionalization strategies have been widely adopted to introduce polar sites into carbon frameworks. In particular, doping with heteroatoms such as N [10], O [15], S [16], P [17], and halogens [18] can effectively modulate the electronic structure of carbon materials, enhance chemical affinity toward LiPSs, and improve catalytic activity for polysulfide conversion [19].

Among emerging carbon-based materials, graphitic carbon nitride (g-C_3_N_4_) has recently garnered increasing interest as a metal-free two-dimensional host material for LSBs [20,21]. Featuring a unique nitrogen-rich heptazine-based conjugated framework, which provides abundant Lewis-basic sites, g-C_3_N_4_ enables strong chemical interactions with lithium polysulfides. In addition, its structural tunability, chemical stability, and metal-free nature render it an appealing candidate for sustainable energy storage applications [22,23]. Prior studies have established that g-C_3_N_4_-based materials can effectively anchor LiPSs and regulate sulfur redox reactions. Nevertheless, pristine carbon nitride is still hindered by intrinsic drawbacks, including a relatively low specific surface area and semiconducting behavior [24], which limit sulfur dispersion and impede electron transport kinetics. These deficiencies ultimately compromise its catalytic efficiency and rate capability in LSBs, underscoring the necessity of further structural and electronic modulation.

In this work, we present a halogen-doping strategy to overcome the aforementioned limitations by developing an iodine-doped carbon nitride (I-CN) material via a one-step annealing process, which serves as a metal-free sulfur cathode host. Compared with pristine CN, the I-CN material exhibits a significantly increased specific surface area (53.05 m^2^ g^−1^), facilitating homogeneous sulfur dispersion and enhanced interfacial contact. More importantly, the incorporation of iodine atoms disrupts the electronic equilibrium within the CN molecular network, leading to reinforced electron delocalization. This electronic modulation not only markedly improves the intrinsic electrical conductivity of carbon nitride but also enhances LiPS adsorption capability and promotes Li_2_S nucleation kinetics, thereby enabling fast and sustainable Li–S redox reactions. As a result, the as-prepared S@I-CN electrode delivers a high discharge capacity of 1341.9 mAh g^−1^ at 0.1C with an activated sulfur utilization of 80.1%, and maintains a remarkable rate capability of 472.7 mAh g^−1^ even at 5C. Encouragingly, a high capacity retention of 66.2% is achieved after 800 cycles, underscoring its excellent long-term cycling stability. This halogen-based heteroatom doping strategy thus not only highlights the critical role of electron delocalization in boosting the electrochemical performance of carbon nitride for Li–S batteries, but also offers new insights into the design of advanced metal-free electrocatalysts for broader energy-related applications.

## 2. Materials and Methods

Preparing CN and I-CN: I-CN was synthesized via a mixed-precursor calcination-exfoliation strategy. Specifically, 6 g of dicyandiamide and 3 g of NH_4_I were thoroughly ground in an agate mortar for 5 min. Subsequently, 20 mL of ethanol was added, and the mixture was stirred and heated at 70 °C for 4 h to obtain a homogeneous solid precursor. After complete solvent removal, the dried precursor was further ground and then transferred into a covered crucible. The sample was calcined in air by heating to 560 °C at a ramping rate of 5 °C min^−1^ and annealed for 4 h, yielding an orange, lightweight solid product. The obtained material was then ground and ultrasonicated to produce I-CN. For comparison, CN was synthesized by directly calcining dicyandiamide under the same thermal treatment conditions and subsequent treatment steps.

## 3. Results

**Figure 1:**
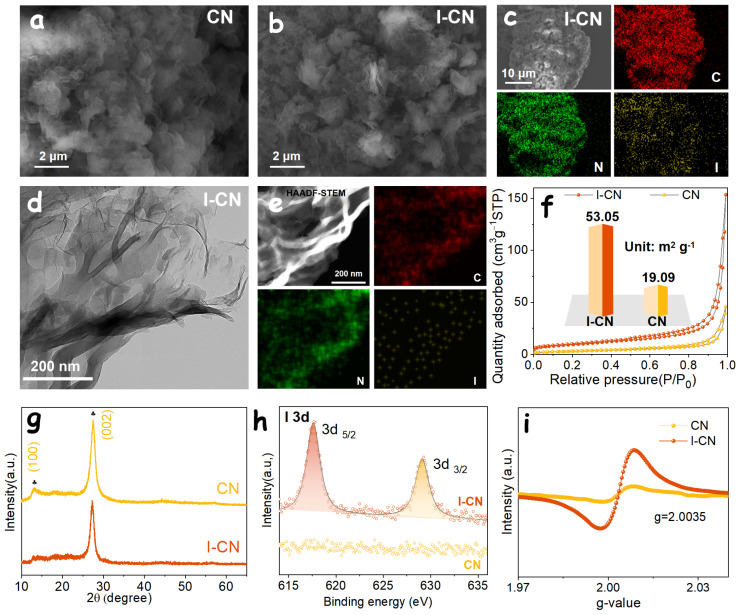
SEM images of the as-prepared (**a**) CN and (**b**) I-CN samples. (**c**) EDS mapping images of the I-CN sample. (**d**) TEM image of the I-CN sample, together with the corresponding (**e**) HAADF-STEM image and EDS mapping results. (**f**) N_2_ adsorption–desorption isotherms, (**g**) XRD patterns, (**h**) I 3d XPS spectra, and (**i**) EDR test results of the I-CN and CN samples.

A precursor regulation strategy was employed to synthesize I-CN alongside pristine CN, in which ammonium iodide was used as the iodine source. Owing to its facile thermal decomposition, ammonium iodide is expected to enable homogeneous incorporation of iodine into the carbon nitride framework. Photographs of the as-prepared samples are shown in Figure S1. Compared with the pale yellow appearance of pristine CN, the I-CN sample exhibits a distinct orange-yellow color, suggesting the successful and uniform doping of iodine. Scanning electron microscopy (SEM) images (Figure 1a,b) reveal that both CN and I-CN possess typical nanosheet-like morphologies. Energy-dispersive X-ray spectroscopy (EDS) elemental mapping further demonstrates the homogeneous distribution of C, N, and I elements within the I-CN sample (Figure 1c), with the iodine content determined to be approximately 3.72 wt% (Figure S2). Transmission electron microscopy (TEM) images further confirm the nanosheet morphology of I-CN (Figure 1d) and its uniform elemental distribution (Figure 1e). For comparison, the elemental distribution of the pristine CN sample was also examined by EDS mapping (Figure S3), indicating a uniform presence of carbon and nitrogen throughout the samples.

The nitrogen adsorption–desorption isotherms of the two samples are presented in Figure 1f. According to the Barrett–Joyner–Halenda (BJH) model, the specific surface area of the I-CN sample reaches 53.05 m^2^ g^−1^, which is significantly higher than that of pristine CN (19.09 m^2^ g^−1^). This enhancement can be attributed to the sublimation and decomposition of NH_4_I into NH_3_ and HI during the calcination process, which promotes the generation of additional porosity and increases the specific surface area during the high-temperature polymerization of dicyandiamide into CN.

X-ray diffraction (XRD) analysis was conducted to elucidate the phase structure of the carbon nitride materials. As shown in Figure 1g, both CN and I-CN exhibit highly similar diffraction patterns. The characteristic peaks located at 12.9° and 27.75° can be indexed to the (100) and (002) planes of typical g-C_3_N_4_, corresponding to the in-plane structural packing and interlayer stacking of g-C_3_N_4_ sheets, respectively. Notably, the (100) diffraction peak intensity of the I-CN sample is markedly weakened compared to that of pristine CN, indicating a reduced degree of in-plane structural ordering. This phenomenon is likely associated with in-plane iodine doping, which disrupts the planar structural stability of the g-C_3_N_4_ framework, in good agreement with previously reported studies [25].

The thermal stability of the I-CN and CN samples was also comparatively evaluated. As shown in Figure S4, the thermogravimetric analysis (TGA) and derivative thermogravimetric (DTG) curves of both samples obtained under an N_2_ atmosphere exhibit highly similar profiles. Both materials maintain excellent thermal stability up to 500 °C and undergo complete thermal decomposition at approximately 750 °C. During the weight-loss process, the maximum decomposition rate occurs in the temperature range of 720–730 °C.

Notably, in contrast to CN, I-CN does not exhibit any additional weight loss peak, effectively ruling out the possibility that iodine-containing species remain in the sample as physically adsorbed residues. However, the temperature corresponding to the maximum weight loss rate of CN is slightly higher than that of I-CN, which can be attributed to the structural destabilization induced by iodine doping. The incorporation of iodine is likely to introduce lattice perturbations that marginally accelerate the thermal decomposition of I-CN, further supporting the successful doping of iodine. Nevertheless, I-CN retains the intrinsically high thermal stability characteristic of carbon nitride-based materials.

X-ray photoelectron spectroscopy (XPS) was further employed to investigate the elemental composition and chemical states of the CN and I-CN samples. The XPS survey spectra indicate that the CN sample is mainly composed of C, N, and O elements, whereas distinct iodine signals are additionally detected in the I-CN sample (Figure S5), confirming the successful incorporation of iodine. The presence of O is likely attributable to adsorbed H_2_O molecules or surface hydroxyl (·OH) species originating from ambient exposure. In the C 1s spectrum (Figure S6a), the peaks located at 284.6, 285.8, and 288.2 eV can be assigned to C–C, C–O, and N–C=N species, respectively [25]. The N 1s spectrum (Figure S6b) can be deconvoluted into four components at 398.3, 399.6, 400.7, and 403.9 eV, corresponding to sp^2^-hybridized C=N–C species, tertiary nitrogen [N–(C)_3_], N–H groups, and oxidized nitrogen species, respectively [26]. A direct comparison of the I 3d spectra (Figure 1h) clearly confirms the presence of iodine in the I-CN sample. The characteristic peaks located at 617.6 and 629.2 eV are assigned to the I 3d_5/2_ and I 3d_3/2_ spin–orbit components, respectively [27]. In contrast, no iodine-related signals are observed in the CN sample, further verifying that iodine is exclusively introduced through the doping process.

Electron paramagnetic resonance (EPR) spectroscopy was further employed to evaluate the influence of the iodine doping on the generation of unpaired electrons in the carbon nitride products. As shown in Figure 1i, both CN and I-CN samples exhibit a symmetric single Lorentzian signal centered at a g value of 2.0035, which is commonly assigned to unpaired electrons localized on sp^2^-hybridized carbon atoms within the heptazine units of g-C_3_N_4_ [27,28]. Notably, the EPR signal intensity of the I-CN sample synthesized using ammonium iodide as the precursor is significantly enhanced compared to that of pristine CN. This pronounced increase indicates a higher concentration of unpaired electrons, suggesting a more effective enhancement of electron delocalization within the CN heterocyclic framework after iodine doping. Such behavior can be attributed to the synergistic effects of increased nitrogen vacancies generated during the rapid decomposition of the NH_4_I precursor and the incorporation of iodine into the carbon nitride matrix, which together promote defect formation and electronic structure modulation.

The above characterization results clearly demonstrate that the co-thermal polymerization process using NH_4_I and dicyandiamide as precursors can achieve effective structure regulation and iodine doping of the carbon nitride framework. During the heat treatment, as the temperature increases, the in situ decomposition of ammonium iodide generates NH_3_ and HI vapor, which not only further optimizes the pore structure of C_3_N_4_ but also enables the in situ release of active iodine species that are chemically introduced into the carbon nitride framework, resulting in a defect-rich structure and facilitating electron delocalization.

**Figure 2:**
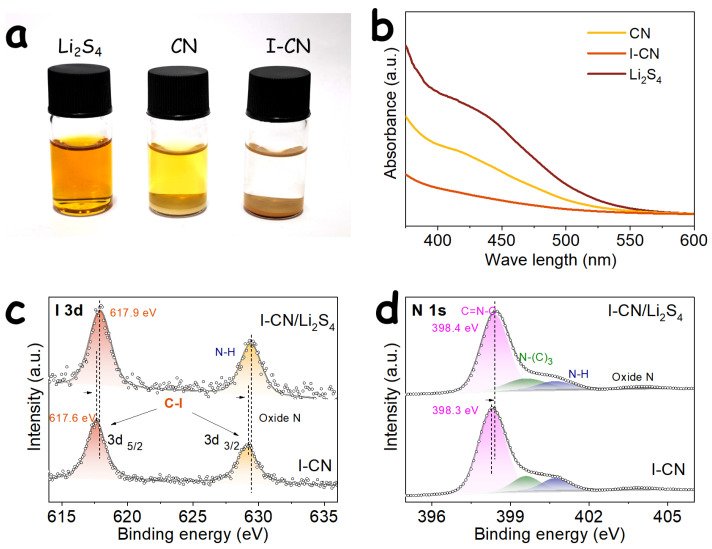
(**a**) Visual comparison of the adsorption behavior of CN and I-CN in a Li_2_S_4_ solution and (**b**) the corresponding UV–vis spectra. (**c**) Comparison of the I 3d and (**d**) N 1s XPS spectra of the I-CN sample before and after adsorption.

Effectively suppressing the polysulfide shuttle effect through rational host material design is critical for improving the cycling stability of LSBs. Accordingly, the adsorption capability of CN and I-CN toward LiPS was evaluated by immersing the materials in Li_2_S_4_ solutions of identical concentration, as illustrated in Figure 2a. After a period of static adsorption, the initially orange-yellow Li_2_S_4_ solution containing the I-CN host becomes nearly colorless, whereas the solution containing CN as the adsorbent retains a noticeable yellow tint. This visual observation indicates a markedly stronger affinity of I-CN toward soluble Li_2_S_4_ species. Such enhanced adsorption can be attributed to multiple factors. On the one hand, the Lewis-basic pyridinic nitrogen atoms inherent to the g-C_3_N_4_ framework, together with the introduced iodine species, can strongly interact with the terminal lithium atoms in lithium polysulfides, which act as Lewis-acidic centers. On the other hand, the significantly increased specific surface area of I-CN facilitates more effective contact between active sites and Li_2_S_4_ molecules.

Ultraviolet–visible (UV–vis) absorption spectroscopy (Figure 2b) was further employed to quantitatively assess the relative concentration of polysulfides in the supernatant solutions. In the characteristic absorption region of Li_2_S_4_ (400–450 nm) [29], the supernatant corresponding to the I-CN adsorbent exhibits the lowest absorbance intensity, indicating that a substantial fraction of Li_2_S_4_ molecules is immobilized by the I-CN host. XPS was subsequently used to probe the changes in chemical environments upon polysulfide adsorption. As shown in Figure 2c, the I 3d_5/2_ and I 3d_3/2_ peaks of the I-CN/Li_2_S_4_ composite display an evident shift toward higher binding energies compared to pristine I-CN, suggesting electron transfer from the iodine dopant sites to the adsorbed Li_2_S_4_ species, with iodine acting as an effective charge-transfer center. Similarly, the N 1s spectrum (Figure 2d) reveals that the characteristic peak of pyridinic nitrogen (C=N–C) shifts noticeably from 398.3 to 398.4 eV after adsorption, which can be ascribed to the anchoring of LiPS at active nitrogen sites via dipole–dipole interactions. These observations are in good agreement with previously reported studies [20].

**Figure 3:**
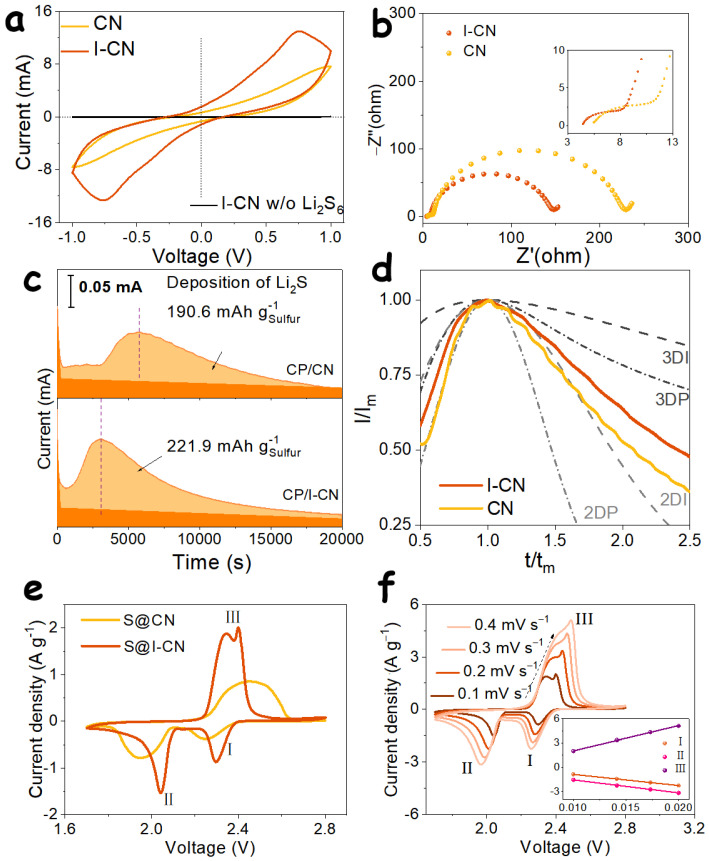
(**a**) CV curves and (**b**) the corresponding Nyquist plots of CN and I-CN in symmetric cells. (**c**) I–t curves of the Li_2_S nucleation process for CN and I-CN, along with (**d**) the corresponding dimensionless model analysis. (**e**) Comparison of the CV curves of S@CN and S@I-CN electrodes at a scan rate of 0.1 mV s^−1^. (**f**) CV curves of the S@I-CN electrode at different scan rates; the inset shows the relationship between the peak currents of the three redox couples and the square root of the scan rate.

Host materials play a crucial role in regulating the multielectron conversion processes of LiPS, thereby exerting a profound influence on the capacity utilization of LSBs. To directly evaluate the reactivity of polysulfide species on different host materials, symmetric cells were employed. As shown in Figure 3a, during cyclic voltammetry (CV) measurements, both CN- and I-CN-based symmetric cells exhibit pronounced redox responses when using a Li_2_S_6_-containing electrolyte, in sharp contrast to the electric double-layer capacitive behavior observed with a blank electrolyte without Li_2_S_6_ (Figure S7). This observation confirms that Li_2_S_6_ is the sole redox-active species in the system. Notably, the I-CN-based symmetric cell delivers significantly higher redox current densities along with a more distinct pair of oxidation and reduction peaks compared to its CN counterpart. These results indicate that the I-CN host effectively promotes the redox kinetics of Li_2_S_6_, reflecting its superior electrocatalytic activity toward polysulfide conversion.

Electrochemical impedance spectroscopy (EIS) provides deeper insight into the solid–liquid interfacial interactions between host materials and soluble electrochemically active species. As shown in Figure 3b, the Nyquist plots of both I-CN- and CN-based electrodes consist of two consecutive semicircles. In the initial region, the smaller intercept on the real axis for the I-CN electrode indicates a lower contact resistance compared to that of CN. In the high-frequency region, both electrodes exhibit a relatively small and depressed semicircle, which is associated with the charge-transfer process at the electrode–electrolyte interface [30]. Notably, the I-CN electrode displays a lower interfacial charge-transfer resistance, reflecting faster redox kinetics. In the middle-to-low frequency region, a larger semicircle is observed for both electrodes, corresponding to the electrochemical conversion of Li_2_S_4_ species at the solid–liquid interface [30]. Owing to its stronger adsorption affinity toward lithium polysulfides and enhanced catalytic activity for polysulfide conversion, the I-CN electrode exhibits a smaller semicircle in this frequency range, further confirming its superior interfacial reaction kinetics.

During the 16-electron multistep conversion process in LSBs, the reduction in soluble Li_2_S_4_ intermediates to the final Li_2_S product via a liquid–solid conversion pathway contributes 12 electrons, accounting for approximately 75% of the total capacity [31]. Therefore, potentiostatic discharge measurements were conducted to investigate the regulatory effect of I-CN and CN samples on Li_2_S nucleation behavior. As shown in Figure 3c, the current–time curves of both samples exhibit a characteristic evolution in which the current gradually increases to a maximum value, followed by a progressive decrease and eventual stabilization. Because the liquid–solid nucleation and conversion of Li_2_S are closely related to the density and activity of intrinsic nucleation sites on the substrate, the larger specific surface area and enhanced electronic delocalization of I-CN endow it with superior catalytic activity, thereby effectively accelerating the nucleation kinetics of Li_2_S. Compared with the CN host, I-CN delivers a markedly higher peak current (0.10 mA for CN vs. 0.14 mA for I-CN) and a substantially shorter peak time (2954 s for I-CN versus 5625 s for CN). Based on Faraday’s law, the Li_2_S nucleation capacity was quantitatively evaluated, revealing that I-CN achieves a nucleation capacity of 221.9 mAh g^−1^, which is significantly higher than that of CN (190.6 mAh g^−1^).

The dimensionless current–time curves obtained by normalizing the nucleation profiles of the two host materials (Figure 3d), together with four classical Li_2_S deposition models, including two-dimensional instantaneous (2DI), two-dimensional progressive (2DP), three-dimensional instantaneous (3DI), and three-dimensional progressive (3DP) nucleation, were used to further elucidate the influence of the host materials on Li_2_S nucleation behavior [32]. During the initial nucleation stage, the CN samples, owing to the relatively low specific surface area and inferior catalytic activity, exhibits nucleation behavior that more closely follows the 2DP model. In contrast, iodine doping in I-CN induces enhanced electronic delocalization, which activates a mixed 2DP–3DP growth mode. After the peak nucleation time, Li_2_S deposition becomes increasingly constrained by surface site passivation, electronic transport efficiency, and Li^+^ diffusion kinetics, leading to a gradual transition of the nucleation behavior toward a mixed two-dimensional instantaneous and three-dimensional progressive growth mode. Clearly, once nucleation sites are progressively passivated, nucleation models with a stronger three-dimensional character are more favorable for continued Li_2_S deposition on the catalyst surface, thereby enabling higher nucleation capacities. In this regard, the I-CN samples exhibit a pronounced advantage over the CN counterpart.

To further elucidate the influence of I-CN and CN as sulfur host materials on the electrochemical performance of LSBs, sulfur was incorporated into the host frameworks via a melt-diffusion process. As shown in Figure S8a, the as-prepared S@I-CN composite exhibits abundant diffraction peaks characteristic of orthorhombic sulfur, which are in good agreement with the standard card (JCPDS No. 08-0247). TGA indicates that the sulfur content in the S@I-CN composite reaches 73.1 wt% (Figure S8b). Furthermore, SEM images combined with EDS elemental mapping reveal a homogeneous distribution of C, N, S, and I throughout the composite, demonstrating that I-CN serves as an effective host for uniformly accommodating sulfur active species. For comparison, the S@CN composite was also prepared under identical conditions, as shown in Figure S9, exhibiting a comparable sulfur loading of 72.6 wt%.

Subsequently, coin cells were assembled using S@CN and S@I-CN composite cathodes paired with lithium foil anodes, and CV measurements were conducted. The corresponding CV curves are shown in Figure 3e. Both cells exhibit two cathodic peaks and one anodic peak. The two reduction peaks are associated with the conversion of S_8_ to soluble long-chain LiPSs (Peak I), followed by their further reduction to solid Li_2_S_2_/Li_2_S species (Peak II) [33]. Conversely, the oxidation peak corresponds to the multistep oxidation of solid Li_2_S_2_/Li_2_S back to long-chain LiPSs and ultimately to solid S_8_ (Peak III) [34]. A direct comparison of the CV profiles reveals that the S@I-CN electrode delivers a substantially stronger current response and higher reduction peak voltage than the S@CN electrode. Specifically, the reduction peak voltage of S@I-CN are located at 2.293 V (Peak I) and 2.041 V (Peak II), which are markedly higher than those of S@CN (2.239 V for Peak I and 1.945 V for Peak II). Meanwhile, the oxidation peak voltage of S@I-CN (2.399 V) is significantly lower than that of S@CN (2.453 V), indicating reduced electrochemical polarization and improved reaction reversibility.

Figure 3f further illustrates the evolution of the CV curves of the S@I-CN electrode at increasing scan rates. As the scan rate increases, the current density correspondingly increases, while the oxidation peak shifts toward higher voltage and the reduction peaks shift toward lower voltage, reflecting the progressive enhancement of electrochemical polarization. Notably, even at a scan rate of 0.4 mV s^−1^, the S@I-CN electrode retains well-defined and complete redox peaks, whereas the S@CN electrode (Figure S10a) exhibits distorted peak shapes at high scan rates due to more severe polarization.

Moreover, the linear relationship between the peak current and the square root of the scan rate for the corresponding redox peaks suggests that the electrochemical reactions of both electrodes belong to diffusion-controlled processes. Thus, the lithium-ion diffusion coefficients during the redox reactions can be quantitatively determined using the classical Randles–sevcik equation [32]:

Ip=(2.69⋅105)n1.5ADLi+CLi+v0.50.5

where *I_p_* is the peak current, *n* is the number of charge transfer, *A* is the geometric cathode electrode area, *D_Li_*_+_ is the *Li*^+^ diffusion coefficient, *C_Li_*_+_ is the concentration of *Li*^+^ in the electrolyte, and *ν* is the scan rate. The linear relationships between the peak current and the square root of the scan rate for the three redox processes of S@CN and S@I-CN are summarized in Figure S10. It is evident that the S@I-CN electrode exhibits consistently steeper slopes than the S@CN electrode, indicating a significantly higher Li+ diffusion coefficient.

Such an enhanced ion diffusion capability is closely associated with accelerated redox kinetics. Taken together with the aforementioned electrocatalytic characterizations, these results reasonably suggest that iodine doping effectively enhances the electronic delocalization of the carbon nitride framework. Consequently, I-CN, acting as a catalytically active sulfur host, substantially promotes the redox kinetics of Li-S reactions and facilitates the efficient conversion of LiPS, thereby contributing to improved electrochemical performance.

**Figure 4:**
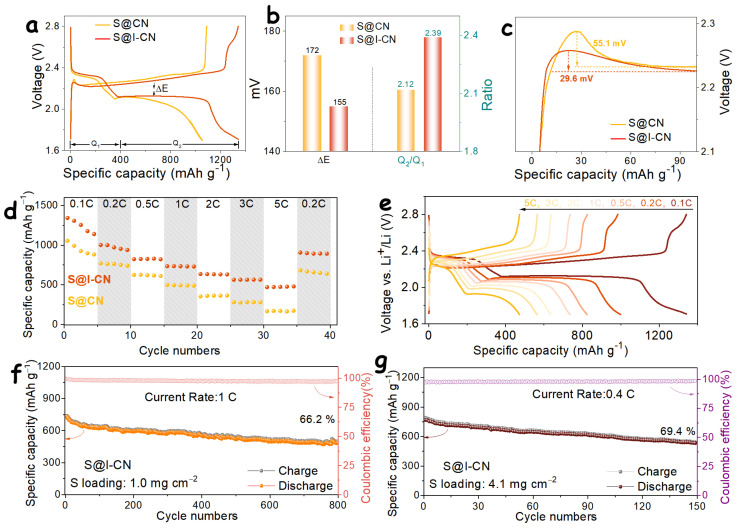
(**a**) Charge–discharge profiles of S@CN and S@I-CN at 0.1C and (**b**) the corresponding ΔE and Q_2_/Q_1_ analyses for the two electrodes. (**c**) Analysis of the Li_2_S oxidation potential during the charging process. (**d**) Rate performance of S@CN and S@I-CN. (**e**) Charge–discharge profiles of the S@I-CN electrode at different current rates. (**f**) Cycling performance of the S@I-CN electrode at 1C and (**g**) cycling stability under high sulfur loading conditions.

Subsequently, galvanostatic charge–discharge measurements were conducted to further evaluate the capacity utilization and cycling behavior of the two electrodes. Figure 4a presents the charge–discharge profiles of S@CN and S@I-CN at a current density of 0.1C. Both electrodes exhibit two distinct discharge plateaus. The high-voltage plateau corresponds to the conversion of solid S_8_ to soluble Li_2_S_4_ species, while the low-voltage plateau is associated with the further reduction in Li_2_S_4_ to Li_2_S_2_/Li_2_S [35]. During the charging process, a single dominant plateau is observed, corresponding to the stepwise oxidation of Li_2_S_2_/Li_2_S back to S_8_, in good agreement with the CV results. Notably, the S@I-CN electrode delivers a high initial discharge capacity of 1341.9 mAh g^−1^, which is significantly higher than that of the S@CN electrode (1055 mAh g^−1^). This improvement demonstrates that the I-CN host effectively enhances sulfur utilization by virtue of its larger specific surface area and superior electrocatalytic activity.

Furthermore, the electrochemical polarization during the charge–discharge process was quantitatively evaluated using the voltage difference (ΔE) between the charge and discharge curves at 50% depth of discharge [36]. As shown in Figure 4b, the S@I-CN electrode exhibits a much smaller polarization voltage (155 mV) compared to that of S@CN (172 mV), indicating reduced electrochemical polarization. In principle, the low-voltage discharge plateau contributes approximately 75% of the total discharge capacity during the Li-S reaction, and thus the capacity ratio between the low- and high-voltage plateaus (Q_2_/Q_1_) is theoretically expected to be 3 [34]. However, due to the formation of intermediate Li_2_S_2_ species and the inevitable polysulfide shuttle effect, the experimentally observed Q_2_/Q_1_ ratio is typically lower than the theoretical value. As shown in Figure 4b, the Q_2_/Q_1_ ratio of the S@I-CN electrode reaches 2.39, which is markedly higher than that of S@CN (2.12). This result suggests that the I-CN host effectively promotes the nucleation and conversion of LiPS to Li_2_S, consistent with the Li_2_S nucleation results discussed in Figure 3c.

With respect to the oxidation of Li_2_S during the initial charging stage, the system must overcome a voltage barrier to activate solid Li_2_S before entering a steady-state charging regime, owing to the intrinsic overpotential required for Li_2_S oxidation [37]. Compared with the S@CN electrode, which requires an overpotential of 55.1 mV, the S@I-CN electrode requires only 29.6 mV. This substantial reduction in overpotential indicates that iodine-doped I-CN effectively lowers the energy barrier for Li_2_S oxidation through enhanced electronic delocalization, thereby accelerating the oxidation kinetics.

Figure 4d compares the rate capabilities of the S@I-CN and S@CN electrodes over a wide range of current rates from 0.1 to 5C. At 0.1C, the S@I-CN electrode delivers a high initial discharge capacity of 1341.9 mAh g^−1^, which is substantially higher than that of the S@CN electrode (1055 mAh g^−1^). With increasing current density, the S@I-CN electrode retains a considerable capacity of 472.7 mAh g^−1^ even at 5C. In contrast, the S@CN electrode exhibits a severely diminished capacity of only 171.9 mAh g^−1^ at the same high rate. When the current rate is returned to 0.2C, the S@I-CN electrode demonstrates excellent capacity recovery, maintaining a high reversible capacity of 906.3 mAh g^−1^, indicative of robust structural integrity and stable electrochemical kinetics.

Figure 4e presents the corresponding charge–discharge profiles of the S@I-CN electrode at various rates. Notably, even at a high rate of 5C, the S@I-CN electrode preserves well-defined dual discharge plateaus, reflecting favorable reaction kinetics and efficient polysulfide conversion. By contrast, the charge–discharge profiles of the S@CN electrode (Figure S11) show a progressive fading of the second discharge plateau with increasing current density due to sluggish reaction kinetics, and the low-voltage plateau becomes nearly indistinguishable at 5C.

The cycling stability of the S@I-CN electrode was further evaluated. As shown in Figure 4f, at a current density of 1C, the S@I-CN electrode retains a high reversible capacity of 483.2 mAh g^−1^ after 800 cycles, corresponding to a capacity retention of approximately 66.2%. This performance translates to an exceptionally low capacity decay rate of only 0.042% per cycle, with an average Coulombic efficiency exceeding 97.6%. The charge–discharge profiles at different cycle numbers are presented in Figure S12. The well-preserved curve shapes throughout prolonged cycling further confirm the excellent cycling stability of the S@I-CN electrode. Table S1 summarizes the recently reported electrochemical performance of metal-free g-C_3_N_4_-based materials in LSBs. In comparison with these previously reported systems, the I-CN host developed in this work exhibits competitive rate capability and cycling stability. Considering that practical LSBs require high areal capacities, the sulfur areal loading was increased to 4.1 mg cm^−2^. Under a current density of 0.4C, the S@I-CN electrode exhibits a capacity retention of 69.4% after 150 cycles, demonstrating robust cycling stability even at high sulfur loadings. As shown in Figure S13, although increased polarization is observed at high areal loading, the S@I-CN electrode maintains stable and reversible capacity output. The high-loading test results further confirm that iodine-doped I-CN effectively promotes the redox conversion of LiPS, thereby enabling stable and high-performance LSB operation.

## 4. Conclusions

This work reports a precursor-regulated strategy for synthesizing I-CN as an advanced LSB host. The incorporation of NH_4_I serves a dual function: it acts as a pore-forming agent to substantially increase the specific surface area, while simultaneously achieving iodine doping that disrupts the in-plane charge balance and induces electron delocalization. This electronic modulation transforms the semiconducting carbon nitride into a material with significantly enhanced intrinsic conductivity. The optimized porous architecture provides abundant sulfur confinement sites and facilitates rapid ion transport, which directly enables the outstanding rate capability, delivering a reversible capacity of 472.7 mAh g^−1^ even at 5C. Furthermore, the combination of high surface area and iodine-enhanced polar sites enables strong chemisorption of lithium polysulfides, effectively mitigating the shuttle effect. This accounts for the stable cycling performance, achieving 69.4% capacity retention after 150 cycles under high sulfur loading. By quantitatively linking the iodine-induced structural and electronic modulations to the enhanced electrochemical kinetics and stability, this work provides a design blueprint for developing efficient, metal-free host materials for advanced sulfur-based batteries.

## Data Availability

The data presented in this study are available on request from the corresponding author.

## Supplementary Materials

The following supporting information can be downloaded at: https://www.mdpi.com/article/10.3390/nano16050291/s1, Figure S1: Digital photographs comparing the as-prepared CN and I-CN samples. Figure S2: Elemental composition of the I-CN sample determined by EDS analysis. Figure S3: SEM images and the corresponding EDS mapping results of the CN sample. Figure S4: Thermogravimetric behavior of CN and I-CN samples under an N_2_ atmosphere. Figure S5: XPS survey spectra of the CN and I-CN samples. Figure S6: (a) C 1s and (b) N 1s XPS spectra of the I-CN sample. Figure S7: CV curves of the I-CN symmetric cell in the absence of Li_2_S_6_ as the active species. The nearly rectangular CV curves indicate that I-CN exhibits electric double-layer capacitance behavior during the redox process. Figure S8: (a) XRD patterns, (b) TGA curves, and (c) EDS mapping images of the S@I-CN composite. Figure S9: (a) XRD patterns and (b) TGA curves of the S@CN composite. Figure S10: (a) CV curves of the S@CN electrode recorded at different scan rates. Linear relationships between the peak current density and the square root of the scan rate for the S@CN and S@I-CN electrodes at (b) Peak I, (c) Peak II, and (d) Peak III redox processes. Figure S11: Charge–discharge profiles of the S@CN electrode at different current rates. Figure S12: Evolution of the charge–discharge profiles of the S@I-CN electrode during cycling. Figure S13: Evolution of the charge–discharge profiles of the S@I-CN electrode during cycling under high sulfur loading conditions. Table S1: Comparison of electrochemical performance of I-CN as host cathode for LSBs with state-of the-art C_3_N_4_-based metal-free materials. Refs. [38,39,40,41,42,43,44,45,46] are cited in the supplementary materials.

## Abbreviations

The following abbreviations are used in this manuscript:

LSBs	lithium–sulfur batteries
LiPS	lithium polysulfides
g-C_3_N_4_	graphitic carbon nitride
I-CN	iodine-doped carbon nitride
EDS	energy-dispersive X-ray spectroscopy
TEM	transmission electron microscopy
BJH	Barrett–Joyner–Halenda
XRD	X-ray diffraction
TGA	thermogravimetric analysis
DTG	derivative thermogravimetric
XPS	X-ray photoelectron spectroscopy
EPR	electron paramagnetic resonance
UV–vis	ultraviolet–visible
CV	cyclic voltammetry
EIS	electrochemical impedance spectroscopy
2DI	two-dimensional instantaneous
2DP	two-dimensional progressive
3DI	three-dimensional instantaneous
3DP	three-dimensional progressive
