# Supplementary material for: Iodine-Doped Carbon Nitride with Enhanced Electron Delocalization as Metal-Free Sulfur Hosts for Stable Lithium–Sulfur Batteries
Source: Nanomaterials (Basel). 2026 Feb 25;16(5):291. doi: 10.3390/nano16050291 (PMC12986240; doi:10.3390/nano16050291)
Supplement: Supplementary file 1 [file nanomaterials-16-00291-s001.zip › nanomaterials-4163048-supplementary.pdf]

Supporting Information

Iodine-Doped Carbon Nitride with Enhanced Electron Delocalization as Metal-free Sulfur Hosts for Stable Lithium–Sulfur Batteries

Xu Yan[1], Ruxin Liao[1], Kaifu Lin[1], Shiman Fan[1], Ren He[2], Chaoqi Zhang[1]*, Hongbing Zhan[1]*

1. College of Materials Science and Engineering, Fuzhou University, Fuzhou 350108, China.
2. Catalonia Institute for Energy Research–IREC, Sant Adria de Besos, Barcelona 08930, Spain.

Experimental section

*Synthesis of S@I-*CN*, S@*CN. Typically, I-CN-nt and CN mixed with sulfur powder (Sigma Aldrich, 99.98%) separately by the weight ratio of 1:3, and then heated the mixture at 155 °C over night in an autoclave under Ar protection.

Materials Characterization: The crystallographic structural information of the as-prepared products was detected by an X-ray diffractometer (XRD, Miniflex 600) with Cu K radiation (λ = 1.5106 Å) operating at 40 kV and 15 mA. The morphology and microstructure of samples were investigated by FESEM (ZEISS Auriga) equipped with an energy-dispersive X-ray spectroscopy (EDS) detector operated at 15-20 kV. High-resolution TEM (HRTEM) and scanning TEM (STEM) studies were carried out using a field emission gun FEI Tecnai F20 microscope at 200 kV with a point-to-point resolution of 0.19 nm. Elemental mapping analysis was conducted via high-angle annular dark-field scanning TEM energy dispersive X-ray spectroscopy (HAADF-STEM-EDX). The chemical composition and the valence states of the products were observed by employing an X-ray photoelectron spectroscopy (XPS, Thermo Fisher Scientific, ESCALAB 250). The content of sulfur in the composites was estimated by TGA ((PerkinElmer Diamond TG/DTA instrument.) experiments under N_2 atmosphere. The specific surface area and analysis of the pore size distribution were obtained from nitrogen adsorption-desorption

isotherms on Gemini VII2390 system. UV-vis absorption spectra were identified by the PerkinElmer LAMBDA 950 UV-vis spectrophotometer. The electron paramagnetic resonance (EPR) analysis was performed on a Bruker EMX-plus-10/12 spectrometer.

Li-S cell assembly and measurements: Li-S battery performance was tested at room temperature in CR2032 coin-type cells. The assembly process, in which lithium foils were used as anode and Celgard 2400 membranes as separators, S@host composites electrode worked as the cathode, was conducted in a glovebox filled with argon. Cathode was obtained by doctoral blade methods. Briefly, S@host composites (S@CN and S@I-CN), conductive carbon (carbon nanotube: Super P=1:1), and PVDF binder (weight ratio = 7:2:1) were well mixed with the weight ratio of 7:2:1, N-methyl pyrrolidone (NMP, 99.5%, Acros Organics) was added to form a black slurry, and then coated it on carbon-coated Al foils and dried at 60 °C overnight. After drying the foil was punched into small disks with a diameter of 12.0 mm. Sulfur loading was about 1.0 mg cm^{-2}. The electrolyte was prepared by dissolving 1.0 M lithium bis(trifluoromethanesulfonyl)imide (LiTFSI) (99%, Acros Organics) into a solution of 1,2-dimethoxy ethane (DME, 99%, Honeywell) and 1,3-dioxolane (DOL, 99.5%, Alfa Aesar) (v/v = 1/1) and containing 0.2 M of $LiNO_3$ (99.98%, Alfa Aesar). For each coin cell, 15 mL g^{-1}_{Sulfur} of electrolyte was used. In order to realize higher sulfur loadings, the mixed slurry was dropped on the carbon-coated Al foil with 4.1 mg cm^{-2} sulfur loading, and the electrolyte was added with the ratio of 12.5 mL g^{-1}_{Sulfur}. The Li-S cells were galvanostatically cycled at a voltage window of 1.7-2.8 V on a Neware BTS4008 battery tester with different current rates, low current activation was conducted before the cycling test. Cyclic voltammetry (CV) measurements were performed on an electrochemical tester (Gamry 1010E) at a scan rate of 0.1-0.4 mV s^{-1} and electrochemical impedance spectroscopy (EIS) tests were conducted with a voltage amplitude of 10 mV in the frequency range 100 kHz to 10 mHz.

Preparation of Li_2S_4 solutions for adsorption test: Sulfur and Li_2S (99.9%, Alfa Aesar) were mixed with the molar ratio of 3:1, and then the powder was poured into appropriate amounts of DME/DOL (volume ratio of 1:1) solution under vigorous magnetic stirring overnight until a dark

brown solution was obtained. 20 mg of CN and I-CN were poured into 3.0 mL 3.5 mM Li_2S_4 solution, respectively. All the steps were conducted under Ar atmosphere.

Li_2S nucleation measurements

For the study of liquid-solid conversion kinetics, Li_2S_8 solution (0.25 M) was prepared by mixing sulfur and lithium sulfide (molar ratio of 7:1) and dissolving the mixture in tetraglyme solution containing 1.0 M LiTFSI under vigorous stirring for 24 h. Precipitation of Li_2S on various reactive surfaces was investigated in 2032 coin-type cells with a PP separator. 20 μL of 0.25 M Li_2S_8 solution was dropped on the carbon paper coated with CN and I-CN catalyst, and 20 μL of 1M LiTFSI in tetraglyme electrolyte was used on the anode. The cells were discharged galvanostatically at 0.112 mA to 2.12 V, and then kept at 2.07 V for Li_2S to nucleate until the current dropped below 10^{-5} A.

Symmetric Cell Assembly and Measurements: Two pieces of the carbon paper loading with 0.5 mg cm^{-2} host materials were used as identical working and counter electrodes with 40 μL of electrolyte containing 0.5 mol L^{-1} Li_2S_6 and 1 mol L^{-1} LiTFSI dissolved in DOL/DME (v/v = 1/1), the CV measurements were performed at scan rate of 10 mV s^{-1}.

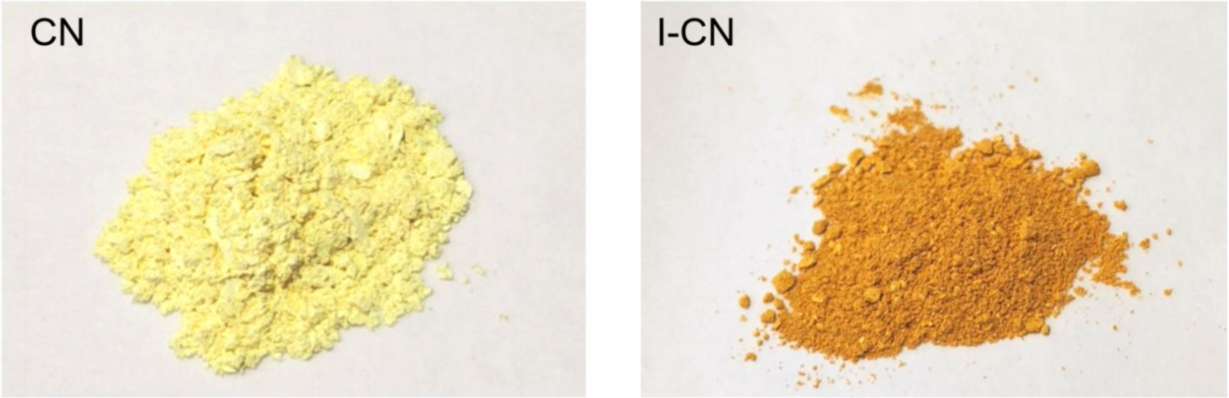

Figure S1. Digital photographs comparing the as-prepared CN and I-CN samples.

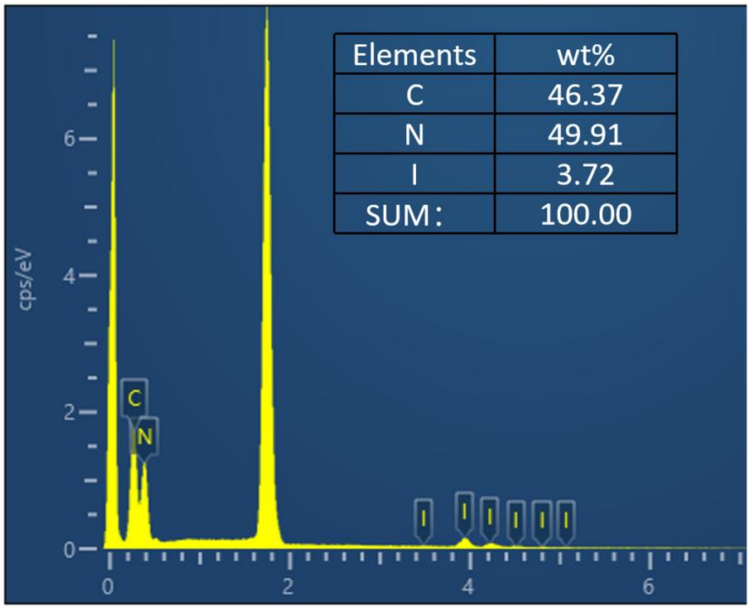

Figure S2. Elemental composition of the I-CN sample determined by EDS analysis.

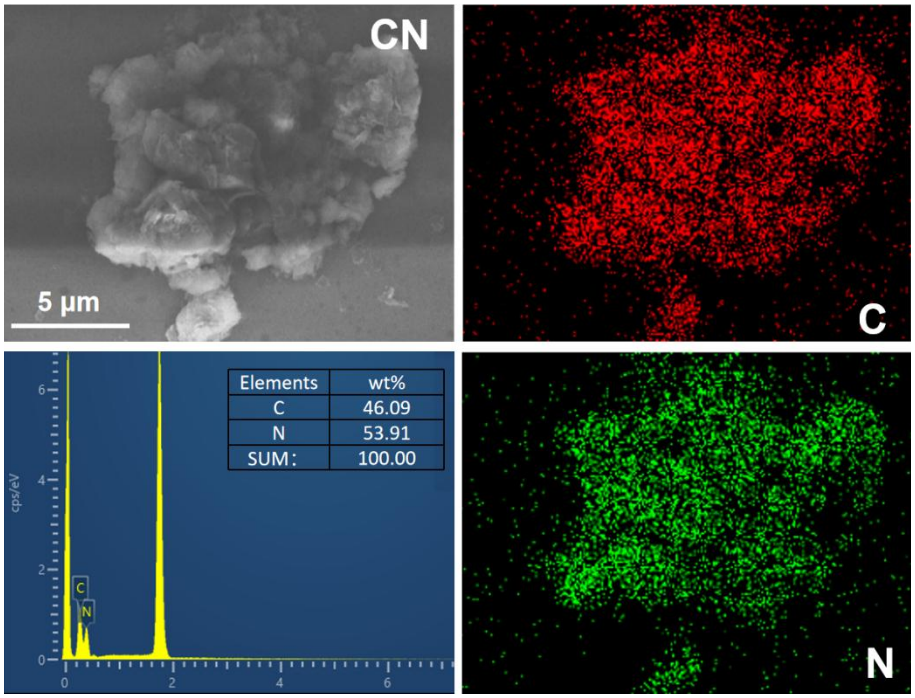

Figure S3. SEM images and the corresponding EDS mapping results of the CN sample.

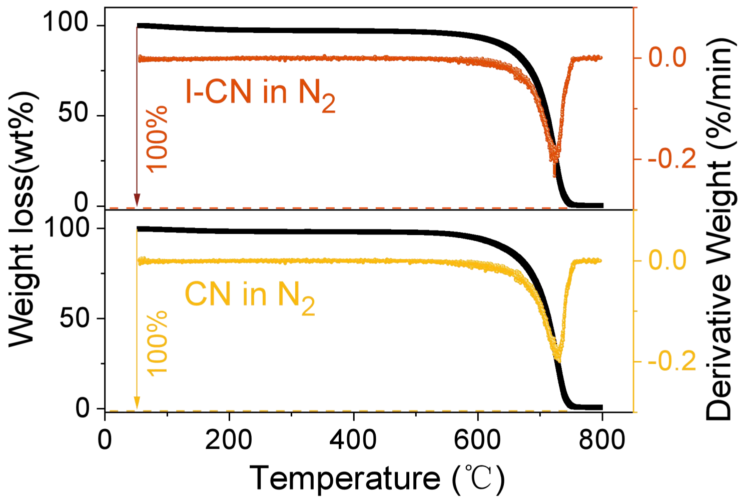

Figure S4. Thermogravimetric behavior of CN and I-CN samples under an N_2 atmosphere.

Note: During the testing process, the N_2 flow rate was maintained at 20 mL/min, with a heating rate of 5 °C/min. The measurement temperature ranged from 50 to 800 °C, and the sample mass used for analysis was approximately 10 mg.

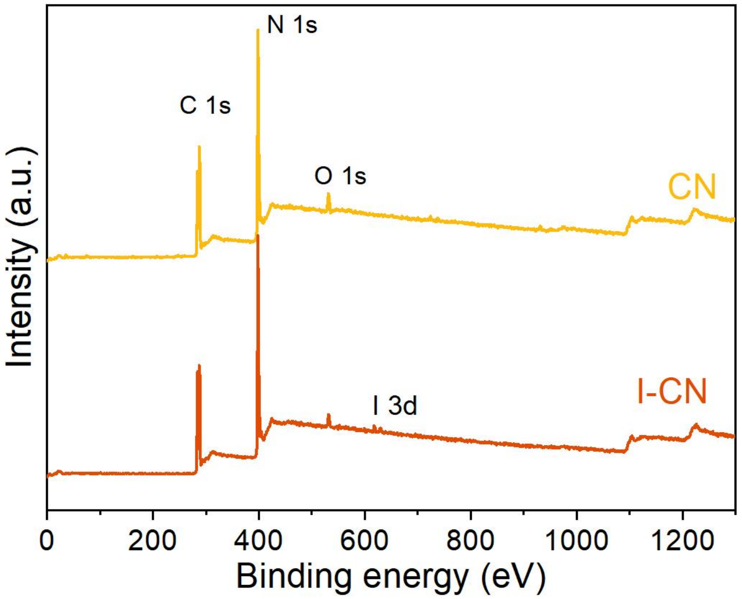

Figure S5. XPS survey spectra of the CN and I-CN samples.

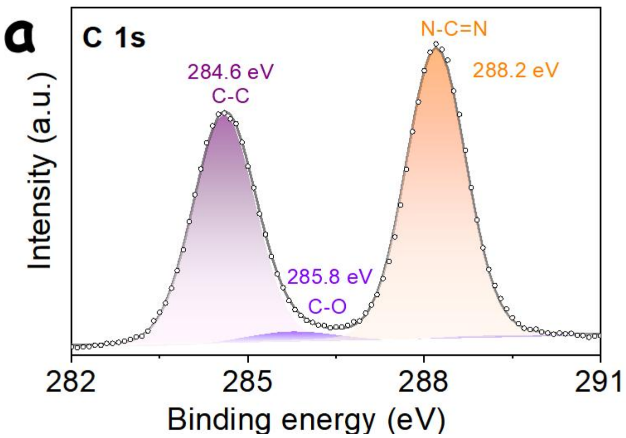

Figure S6. (a) C 1s and (b) N 1s XPS spectra of the I-CN sample.

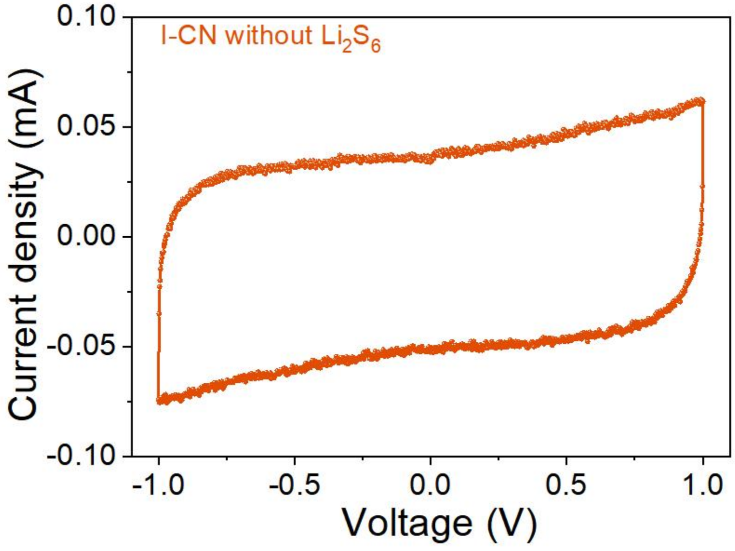

Figure S7. CV curves of the I-CN symmetric cell in the absence of Li2S6 as the active species. The nearly rectangular CV curves indicate that I-CN exhibits electric double-layer capacitance behavior during the redox process.

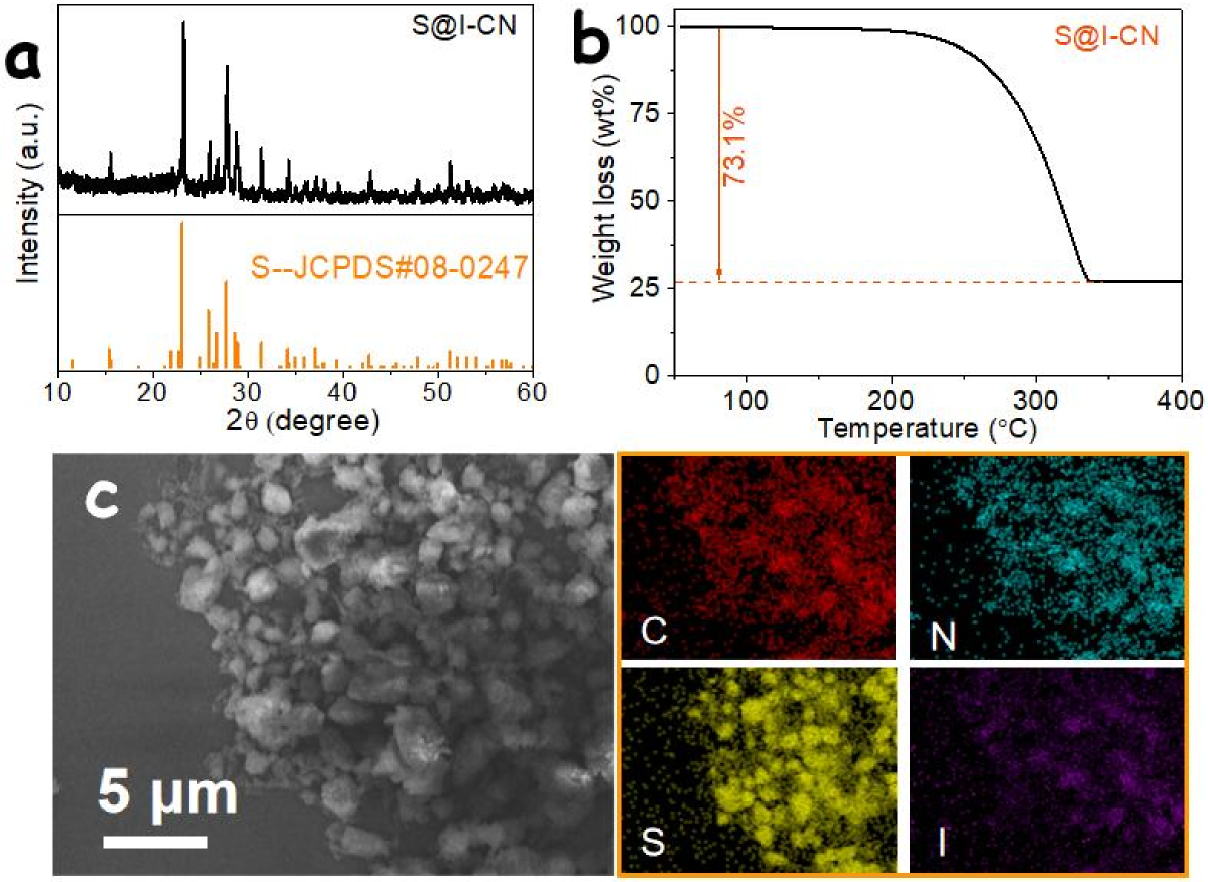

Figure S8. (a) XRD patterns, (b) TGA curves, and (c) EDS mapping images of the S@I-CN composite.

Note: During the TG testing process, the N_2 flow rate was maintained at 20 mL/min, with a heating rate of 5 °C/min. The measurement temperature ranged from 55 to 400 °C, and the sample mass used for analysis was approximately 10 mg.

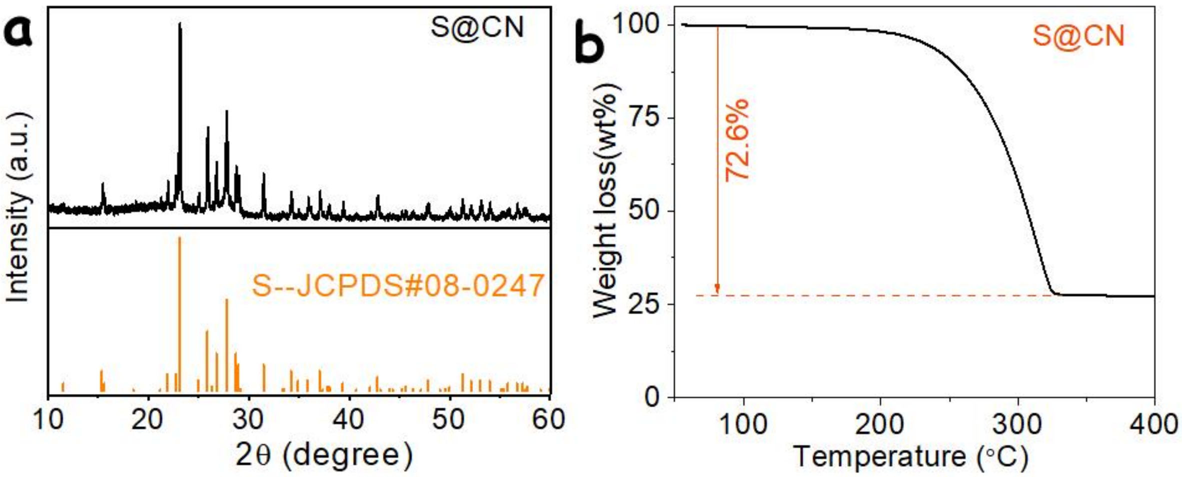

Figure S9. (a) XRD patterns and (b) TGA curves of the S@CN composite.

Note: During the TG testing process, the N_2 flow rate was maintained at 20 mL/min, with a heating rate of 5 °C/min. The measurement temperature ranged from 55 to 400 °C, and the sample mass used for analysis was approximately 10 mg.

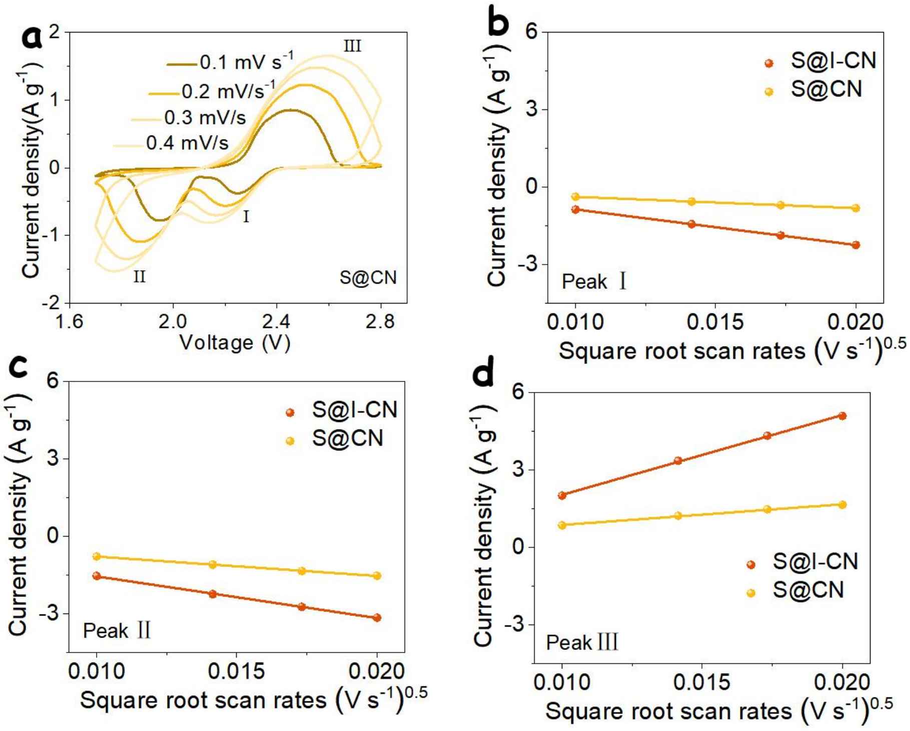

Figure S10. (a) CV curves of the S@CN electrode recorded at different scan rates. Linear relationships between the peak current density and the square root of the scan rate for the S@CN and S@I-CN electrodes at (b) Peak I, (c) Peak II, and (d) Peak III redox processes.

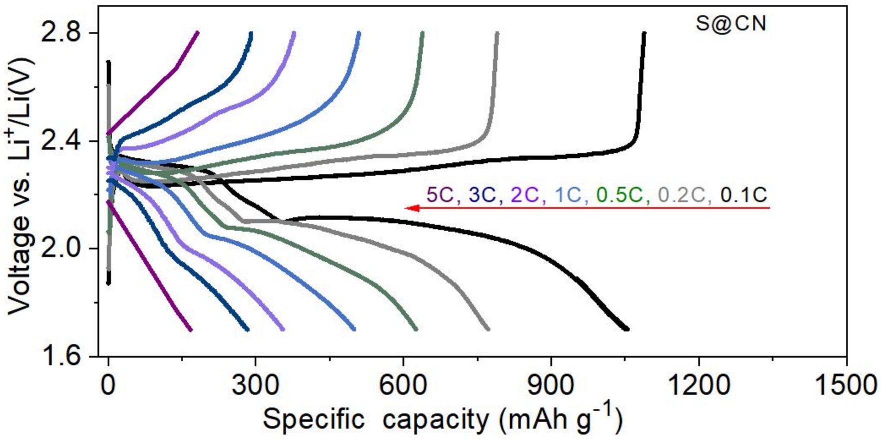

Figure S11. Charge–discharge profiles of the S@CN electrode at different current rates.

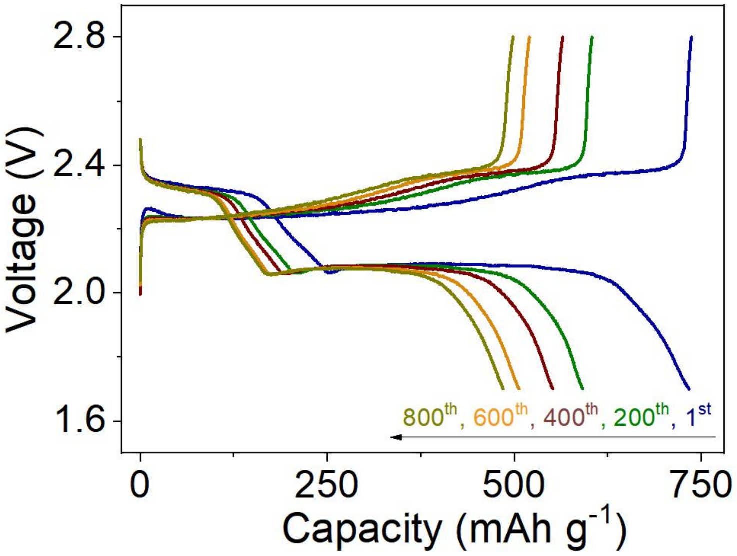

Figure S12. Evolution of the charge–discharge profiles of the S@I-CN electrode during cycling.

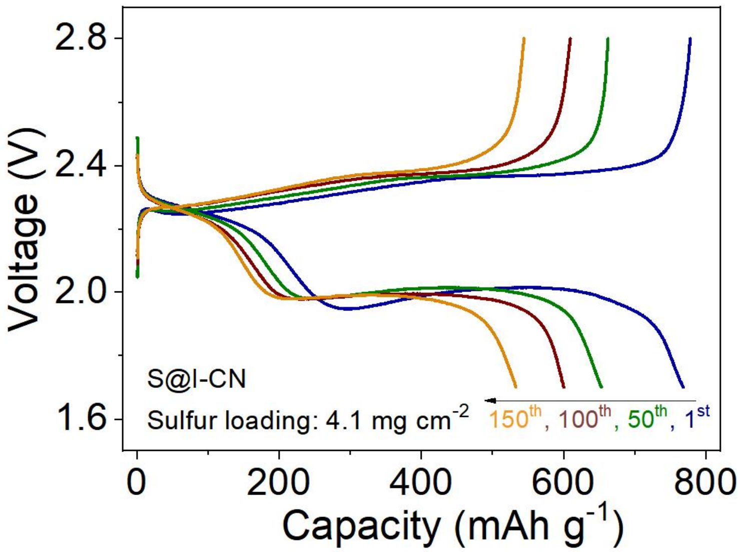

Figure S13. Evolution of the charge–discharge profiles of the S@I-CN electrode during cycling under high sulfur loading conditions.

Table S1. Comparison of electrochemical performance of I-CN as host cathode for LSBs with state-of the-art C_3N_4-based metal-free materials.

C_3N_4-based materials (function)	S content (wt%)	Capacity (mAh g^{-1}) (low/high current rate)	Cycling stability(%) (cycles, current rate)	Decay rate (per cycle, %)	Ref
CNT@NC@GCN (host)	62.5%	1,200 (0.1C) 869.8 (2C)	~59.5% (450 cycles, 1C)	0.09%	[1]
NSPCF@CN-10 (host)	76.1%	1633.0 (0.1C) 976.9 (2C)	~54% (1000 cycles, 1C)	0.046%	[2]
ut-CN (host)	88%	1018 (0.1C) 640 (1C)	~65% (500 cycles, 2C)	0.07%	[3]
CNTs/C_3N_4-10 (host)	70%	1074.3 (0.1C) 510.1 (2C)	73.0% (650 cycles, 0.5C)	0.042%	[4]
C_3N_4/rGO@PPy (host)	70%	1403 (0.1C) 479 (2C)	91% (200 cycles, 1C)	0.045%	[5]
PCNG (interlayer)	80%	1192 (0.1C) 554 (3C)	~60% (800 cycles, 1C)	0.050%	[6]
pCN/CNTsCC1:1 (host)	60%	1201 (0.5C) 553.3 (4C)	60% (500 cycles, 1C)	0.08%	[7]
HDNi@*g*-C_3N_4 (host)	72%	1271.6 (0.1C) 571.96 (2C)	53% (500 cycles, 1C)	0.733%	[8]
g-C_3N_4/CNT (interlayer)	72%	~1300 (0.1C) 755.5 (2C)	~85% (500 cycles, 1C)	0.03%	[9]
**I-CN**	**73.1%**	**1341.9 (0.1 C) 472.7 (5 C)**	**66.2% (800 cycles, 1C)**	**0.042%**	**Our work**

References

1. Chu, F.; Yu, M.; Jiang, H.; Mu, J.; Li, X. Increasing N Active Sites by In-Situ Growing Conformal C3N4 Layer in Hierarchical Porous Carbon-Based Networks for Fast Li+ Transfer and Polysulfide Anchoring in Lithium-Sulfur Batteries. *Journal of Colloid and Interface Science* **2022**, *627*, 838–847,

doi:10.1016/j.jcis.2022.07.113.
2. Chen, Y.; Wu, Y.; Li, L.; Liao, Y.; Luo, S.; Wu, Y.; Qing, Y. Polysulfides Manipulation: Constructing g-C3N4 Networks Encapsulated into Natural Wood Fibers for High-Performance Lithium–Sulfur Batteries. *Chemical Engineering Journal* **2023**, *461*, 141988, doi:10.1016/j.cej.2023.141988.
3. Zou, H.; Zou, Y.; Lv, Y.; Ao, Z.; Chen, N.; Huang, Y. G-C3N4/g-C3N4 Heterojunction as the Sulfur Host for Enhanced Cyclic Stability of Li–S Batteries. *ACS Appl. Energy Mater.* **2022**, *5*, 10067–10075, doi:10.1021/acsaem.2c01683.
4. Cai, X.; Xie, J.; Guo, X.; Zheng, X.; Liu, Y.; Cao, F.; Dong, X.; Kong, Q.; Zhang, J. Suppressing Polysulfide Shuttle in Lithium-Sulfur Batteries Using CNTs/C3N4/S Cathodes. *Materials Today Communications* **2023**, *35*, 106138, doi:10.1016/j.mtcomm.2023.106138.
5. Moon, S.-H.; Shin, J.-H.; Kim, J.-H.; Jang, J.-S.; Kim, S.-B.; Park, Y.-Y.; Lee, S.-N.; Park, K.-W. Polypyrrole Coated G-C3N4/rGO/S Composite as Sulfur Host for High Stability Lithium-Sulfur Batteries. *Materials Chemistry and Physics* **2022**, *287*, 126267, doi:10.1016/j.matchemphys.2022.126267.
6. Zhang, H.; Liu, Q.; Ruan, S.; Ma, C.; Jia, X.; Qiao, W.; Ling, L.; Wang, J. *In-Situ* Construction of *g*-C3N4/Carbon Heterostructure on Graphene Nanosheet: An Efficient Polysulfide Barrier for Advanced Lithium-Sulfur Batteries. *Applied Surface Science* **2022**, *578*, 152022, doi:10.1016/j.apsusc.2021.152022.
7. Wang, W.; Dong, W.; Hong, X.; Liu, Y.; Yang, S. Preparation of G-C3N4/CNTs Composite by Dissolution-Precipitation Method as Sulfur Host for High-Performance Lithium-Sulfur Batteries. *Materials Chemistry and Physics* **2022**, *283*, 126014, doi:10.1016/j.matchemphys.2022.126014.
8. Liu, W.-W.; Niu, S.-T.; Xu, Z.-Q.; Zou, R.; Cui, C.-Y.; Lei, Y.-X.; Zhang, X.-B.; Ran, F. Highly-Dispersed Nickel on 2D Graphitic Carbon Nitrides (*g*-C3N4) for Facilitating Reaction Kinetics of Lithium-Sulfur Batteries. *Applied Surface Science* **2023**, *609*, 155327, doi:10.1016/j.apsusc.2022.155327.
9. Wang, X.; Li, G.; Li, M.; Liu, R.; Li, H.; Li, T.; Sun, M.; Deng, Y.; Feng, M.; Chen, Z. Reinforced Polysulfide Barrier by G-C3N4/CNT Composite towards Superior Lithium-Sulfur Batteries. *Journal of Energy Chemistry* **2021**, *53*, 234–240, doi:10.1016/j.jechem.2020.05.036.
